# The glove hand: an innovative method for effective bedside preoperative flap planning in hand injuries

**DOI:** 10.1308/rcsann.2023.0068

**Published:** 2024-09-01

**Authors:** J Kamath, S S Baliga, L Leo

**Affiliations:** ^1^ Department of Orthopaedics, Kasturba Medical College, Mangalore, Manipal Academy of Medical Sciences (MAHE), Manipal, Karnataka, India; ^2^ Department of Orthopaedics, Father Muller Medical College, Mangalore, Karnataka, India

## Background

Preoperative planning for the use of flaps to treat hand injuries is challenging and requires a thorough understanding of vascular and surface anatomy, as well as competent surgical expertise. We describe an easy novel method for the bedside preoperative planning of flaps used in the treatment of hand injuries.

## Technique

**Figure 1:**
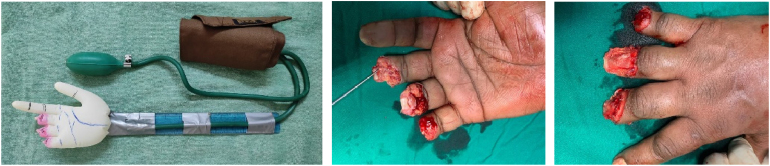
Construct and original hand defect

**Figure 2:**
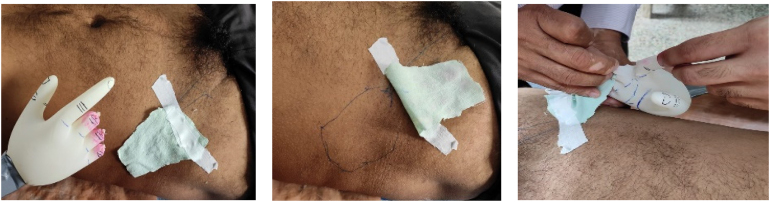
Bedside use of the construct. The area for flap is marked using a piece of lint and a marker.

**Figure 3:**
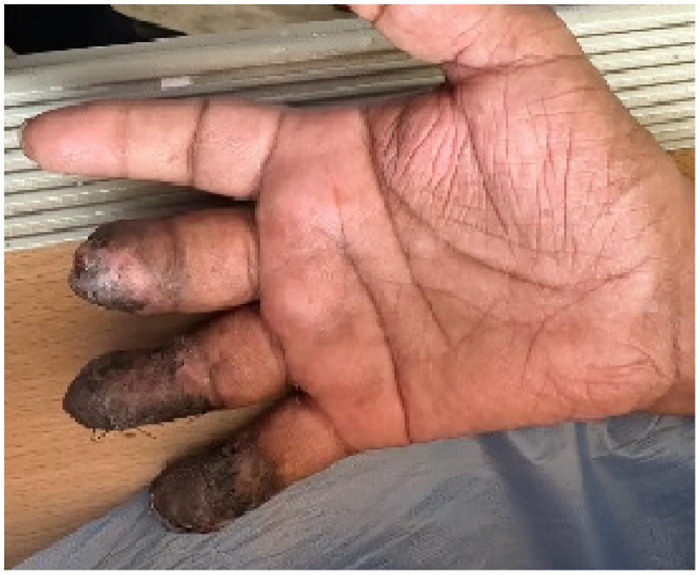
Successfully operated hand

The construct is made up of a surgical glove (recreated to mimic the injured hand) fastened to the tubing of a cuffed blood pressure apparatus, which is strapped to a 30cm ruler to resemble a wrist and forearm model (Figure 1). The glove is inflated using the cuff. The construct is used at the patient's bedside as shown to enable the surgeon to preoperatively determine the type and area of the abdominal or groin flap required to cover the defect (Figure 2). A successful outcome is shown in Figure 3.

## Discussion

This is a simple patient- and surgeon-friendly method for planning flaps. Our construct allows bedside determination of the position of the patient’s wrist and forearm that must be maintained for the required postoperative duration. We note that patients found it easy to understand the surgical procedure when it was illustrated using this method. We also found it academically useful for the teaching of young surgeons.

